# Potential Applications of Clay-Based Therapy for the Reduction of Pesticide Exposures in Humans and Animals

**DOI:** 10.3390/app9245325

**Published:** 2019-12-06

**Authors:** Meichen Wang, Timothy D. Phillips

**Affiliations:** Veterinary Integrative Biosciences Department, College of Veterinary Medicine and Biomedical Sciences, Texas A&M University, College Station, TX 77843, USA

**Keywords:** pesticides, acid activation, adsorption, isotherms, hydra, montmorillonite, clay, enterosorbent

## Abstract

The risk of pesticide exposure in humans and animals may be magnified following natural and man-made disasters such as hurricanes and floods that can result in mobilization and redistribution of contaminated sediments. To develop broad-acting sorbents for mixtures of diverse toxins, we have processed calcium and sodium montmorillonite clays with high concentrations of sulfuric acid. These acid-processed montmorillonite clays (APMs) have shown limited hydration and swelling in water, higher surface areas, and lower trace metal levels than the parent clays, prior to processing. Isothermal analyses have indicated that newly developed APMs are highly active sorbents, with significantly increased binding capacities for a wide range of pesticides, including pentachlorophenol (PCP), 2,4,6-trichlorophenol (2,4,6-TCP), lindane, diazinon, linuron, trifluralin and paraquat. The safety and protective effects of APMs, against pesticide design mixtures, were confirmed in a living organism (*Hydra vulgaris*). Further work is planned to confirm the safety of the APMs in long-term rodent studies. This is the first report of a sorbent material (other than carbon) with high binding efficacy for mixtures of these pesticides. Based on our results, APMs (and similar clays), may be able to decrease human and animal pesticide exposures during disasters and emergencies.

## 1. Introduction

More than 1 billion pounds of pesticides are used in the United States each year and nearly 6 billion pounds are used worldwide [1]. The use of pesticides is of major importance to agriculture, but their widespread occurrence and persistence in the environment can be hazardous to living organisms. It was reported that less than 1% of the total of applied pesticides reach the targets, so a high percentage of these chemicals may move off-target [2,3]. The residues of pesticides are frequently detected in various environmental matrixes, such as soil, water, air, and organism bodies [4]. Moreover, natural and man-made disasters (such as hurricanes and floods) can significantly mobilize these environmental contaminants, exposing humans and animals to contaminated soil and sediment and threatening the safety of municipal drinking water and food sources.

Highly toxic and widely distributed pesticides that are on a watch list due to a potentially severe and/or cumulative risk to human health and/or the environment, or have been banned for use as suggested by the United Nations and the Food and Agriculture Organization [5,6], were chosen for this study. These include: the organochlorines: pentachlorophenol (PCP), 2,4,6-trichorophenol (2,4,6-TCP) and lindane; an organophosphate: diazinon; a urea-type pesticide: linuron; a dinitroaniline: trifluralin; and a bipyridyl pesticide: paraquat [7].

PCP is widespread, persistent in the environment and highly toxic to humans and animals [8]. It has been classified as a possible carcinogen by the International Agency for Research on Cancer (IARC) [9]. PCP has been banned for purchase and use by the general public, but it is still used frequently for industrial applications. 2,4,6-TCP has been commonly used as a pesticide and wood preservative [10]. It has been reported that exposure to 2,4,6-TCP may increase the risk of behavioral impairment in children and carcinogenicity in humans. These chlorophenol compounds are persistent in the environment and can be commonly detected in rivers, ponds and soils [11]. Lindane is a hexachlorocyclohexane that is widely used to treat scabies and pediculosis. It is persistent and undegradable, and thus tends to bioaccumulate in the food chain [12].

Diazinon is an organophosphorus insecticide, which has been effectively used throughout the world with applications in agriculture and horticulture for controlling insects. Its toxicity is due to the inhibition of the enzyme acetylcholine esterase [13].

Linuron is a phenylurea herbicide used widely to selectively control weeds and grasses by inhibiting photosynthesis. It has been shown to act as an androgen receptor ligand that can competitively inhibit the binding of androgens and produce reproductive malformations [14,15].

Trifluralin is dinitroaniline herbicide which controls a wide variety of grasses and broadleaf weeds by interrupting mitosis, controlling weeds as they germinate. It is one of the most widely used herbicides [16]. Trifluralin has been banned in the European Union since 2008, primarily due to its high toxicity to aquatic life [17].

Paraquat is one of the most widely used herbicides due to its rapid, contact-dependent killing of weeds and plants [18]. It is the leading cause of death from pesticide self-poisoning and murder.

**Figure 1.**
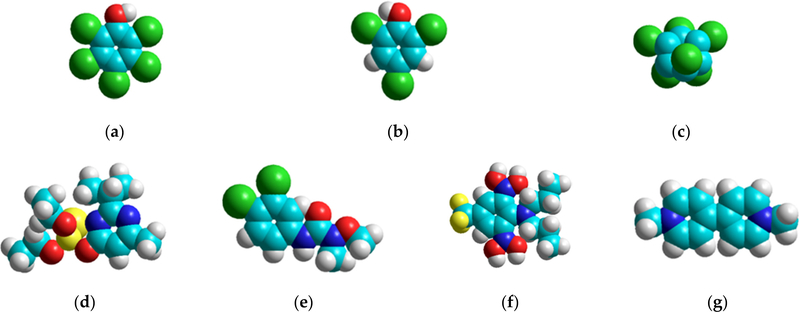
Molecular models of: (**a**) pentachlorophenol (PCP); (**b**) 2,4,6-trichorophenol (2,4,6-TCP); (**c**) lindane; (**d**) diazinon; (**e**) linuron; (**f**) trifluralin; (**g**) paraquat, illustrating the spatial orientation and size of the functional groups.

Exposure to these pesticides can stimulate lipid peroxidation, paralyze the respiratory system, cause endocrine disruption and affect the nervous and reproductive system, etc. [19–21]. The molecular models of each pesticide, derived from a computational quantum mechanical AM1 method in HyperChem, are shown in Figure 1.

Previously, our laboratory has conducted a series of intervention studies showing that montmorillonite clay is safe for short-term human and animal consumption [22]. The idea of including clay-based sorbents in the diet has been fueled by the historical perspective that clay minerals and other sorbent materials have been commonly used as ancient medicine for humans and animals since earliest recorded history. The hypothesized mechanism for this protection involves adsorption of toxins onto active surfaces of sorbents, resulting in reduced concentration of toxin in the gastrointestinal tract and decreased bioavailability and toxicity [23]. To develop broad-acting sorbents to mitigate pesticide exposures and toxicities, we have treated both calcium and sodium montmorillonite clays (CM and SM) with 12 and 18 normality sulfuric acid to produce high surface area and enhanced porosity. These acid-processed calcium and sodium montmorillonites (APCMs and APSMs) have been previously reported to bind hazardous mixtures of mycotoxins (aflatoxin and zearalenone), a commonly occurring herbicide (glyphosate) and polychlorinated biphenyls (PCBs) [24,25]. Based on earlier work with acid processed montmorillonite clays (APMs), we have postulated that treatment of clays with high concentrations of acid results in the exchange of interlayer cations with protons from the acid, followed by the dissolution of portions of the octahedral and tetrahedral framework in the clay structure [26]. The final reaction product of acid treatment is postulated to be a mixture of delaminated parent clay with amorphous silica chains at the edges and porous amorphous silica formed from three-dimensional cross-linked SiO_4_ [25,27]. Importantly, parent montmorillonite clays which were used as starting materials for the formation of APMs are safe for human and animal consumption [22].

This study has focused on the application of broad-acting sorbents (acid-processed montmorillonite clays) for the binding of hazardous pesticides and pesticide mixtures. We have investigated the binding parameters of APMs with equilibrium isotherms and used hydra analysis to predict the ability of clay treatment to prevent the adverse effects of toxin mixtures.

## 2. Materials and Methods

### 2.1. Reagents

High-performance liquid chromatography (HPLC)-grade methanol, acetonitrile and pH buffers (4.0, 7.0 and 10.0) were purchased from VWR (Atlanta, GA, USA). Analytical standards for lindane, diazinon, linuron, trifluralin, paraquat and ammonium acetate were purchased form Sigma Aldrich (St. Louis, MO, USA). Sulfuric acid (H_2_SO_4_, 95–98%), PCP and 2,4,6-TCP were purchased from Aldrich Chemical Co. (Milwaukee, WI, USA). Ethylene glycol and formic acid (HCOOH, 88%) were purchased from Thermo Fisher (Waltham, MA, USA). CM was obtained from BASF (Lampertheim, Germany) with a total surface area of approximately 850 m^2^/g, an external surface area of 70 m^2^/g and cation exchange capacity equal to 97 cmol/kg [28]. SM clay was obtained from the Source Clay Mineral Repository at the University of Missouri-Columbia with an estimated cation exchange capacity equal to 75 cmol/kg. The generic formula for these clays is: (Na,Ca)_0.3_(Al,Mg)_2_Si_4_O_10_(OH)_2⋅_nH_2_O. Clays were sieved through 45 μm to achieve uniform particle size [28,29]. Ultrapure deionized water (18.2 MΩ) was generated in the lab using an Elga™ automated filtration system (Woodridge, IL, USA) and used in all experiments.

### 2.2. Synthesis of Sorbents

Synthesis of APMs was previously described [25]. Briefly, CM and SM were treated with sulfuric acid at two different concentrations (12 and 18N). The solutions were vigorously stirred and kept in an oven at 60 °C overnight. The slurry was cooled, centrifuged at 2000 *g* for 20 min and washed thoroughly with distilled water. This centrifugation-washing process was repeated multiple times until the pH for each group was constant. All samples were dried in the oven at 110 °C overnight before grinding and sieving through 125 μm mesh before use.

### 2.3. Coefficient of Linear Expansibility in Water

Sorbent samples were added to the 2 mL mark in graduated cylinders, and then stirred with 15 mL of water. After 24 h, following thorough equilibrium hydration and swelling, the final sorbent volume was determined. The ratio calculated from the beginning (2 mL) and final volumes was indicative of hydration and expansion of the sample. A higher ratio indicated greater hydration and expansion of the sample [30].

### 2.4. Surface Area Determination

The total surface areas of CM and APCMs were determined by ethylene glycol (EG) [28,31]. EG is retained on the solid surface at monolayer coverage under an applied vacuum of approximately 0.1 mm Hg. The surface area was calculated based on the following equation

(1) A=Wa/(Ws×EGconversionfactor)

where A is the total surface area (m^2^/g), Ws is the oven-dry weight of the clay (g), and Wa is the weight of EG retained by the clay (g). The conversion factor for EG is 3.1 × 10^−4^ g/m^2^ [31].

### 2.5. In Vitro Isothermal Adsorption

The toxin solutions of PCP, 2,4,6-TCP and diazinon were individually prepared by dissolving pure crystals into distilled water at pH 2 to simulate stomach pH. Final solutions were equal to 4 ppm (μg/mL) PCP, 6 ppm 2,4,6-TCP, and 10 ppm diazinon. Paraquat was dissolved into distilled water at pH 7 (since its structure is unaffected by pH) with a final solution equal to 5 ppm paraquat. Other toxin solutions were dissolved into the individual mobile phase (based on the methods of detection) with 12.5 ppm lindane (acetonitrile:water, 50:50), 20 ppm linuron (acetonitrile:water, 65:35) and 20 ppm trifluralin (acetonitrile:water, 70:30). The maximum concentrations were set based on the octanol-water partitioning coefficients (K_ow_) so that precipitation was not a factor, and the optimal ratio of toxin/clay to reach saturation (equilibrium) on isotherm plots was investigated. Then a concentration of 0.002% of sorbent was exposed to an increasing concentration gradient (5–100%) of toxin solution. In these studies, controls consisted of untreated solution, toxin solution without sorbent and sorbent suspension without toxin. The control and test groups were capped and agitated at 1000 rpm for 2 h at ambient temperature (24 °C) using an IKA® electric shaker (VIBRAX VXR basic, Werke, Germany). This time was based on preliminary data suggesting that equilibrium of the surface interaction was reached within 30 min. All samples were then centrifuged at 2000 *g* for 20 min to separate the clay/toxin complex from solution and were detected by either ultraviolet (UV)/visible scanning spectrophotometry, HPLC, or liquid chromatography/tandem mass spectrometry LC/MS/MS.

A SHIMADZU UV/visible scanning spectrophotometry (UV-1800, SHIMADZU Corporation, Kyoto, Japan) was used to analyze PCP and 2,4,6-TCP concentrations in the resulting supernatants for each sample [32–34]. The concentrations were determined in supernatant samples that were placed in a quartz cuvette versus a blank and scanned through the UV region of the electromagnetic spectrum (between 200 and 800 nm) to establish the wavelength for maximal absorption of PCP and 2,4,6 TCP. The concentrations of toxins were determined at 210 nm (PCP) and 294 nm (2,4,6-TCP).

HPLC (Milford, MA, USA) with a Phenomenex® luna 5u C18 column (250 × 4.6 mm) kept at an ambient temperature, was used to measure the absorption of lindane and linuron [35,36]. Lindane was separated by 50% acetonitrile and 50% water as the mobile phase at 2.0 mL/min flow rate and 10 μL injection volume. Free lindane concentration in the supernatant was detected by a UV detector at 254 nm wavelength [35]. For linuron, separation was achieved by a mobile phase of 65% acetonitrile and 35% water, a flow rate at 1.0 mL/min, 20 μL injection volume and UV absorption at a 210 nm wavelength [36]. Breeze® software was used to control the HPLC system and collect data.

HPLC with a SUPELCOSIL LC-18 column (15 × 4.6 mm, 3 μm) was used for detection of trifluralin in the supernatant [37]. Trifluralin analysis was conducted using 70% acetonitrile and 30% water as mobile phase at a flow rate of 1.5 mL/min. The column was maintained at 30 °C and the injection volume was 10 μL. Trifluralin detection was programmed at 254 nm wavelength by the UV detector. Breeze® software was used to control the HPLC system and collect data.

Diazinon and paraquat were analyzed using a Waters Acquity® ultra performance LC/MS/MS (Milford, MA, USA) equipped with triple quadrupole and methods previously described [38,39]. For diazinon, an Acquity® BEH C18 column (2.1 × 50 mm, 1.7 μm) was used and kept at 35 °C. A gradient elution using 10 mM ammonium acetate (eluent A) and 10 mM ammonium acetate in methanol (eluent B) was carried out (eluent B, 10–90% linear gradient for 8 min) at a flow rate of 0.6 mL/min. A sample volume of 5 μL was used for each analysis. MS analysis was performed with an electrospray ionization (ESI) interface and operated in a positive ion mode. The spray voltage was maintained at 5 kV. The source temperature was kept at 500 °C. The mass spectrometer was monitored at *m*/*z* 305.1 to 169.2 for precursor and product ions of diazinon [38]. For paraquat, LC/MS/MS with a hydrophilic interaction liquid chromatography (HILIC) column (2.1 × 100 mm, 3 μm) at 30 °C was used to determine its concentration. Separation using a mobile phase containing 10 mM ammonium acetate with 0.1% formic acid (eluent A) and acetonitrile (eluent B) was carried out at a flow rate of 0.2 mL/min and injection volume of 10 μL. The following gradient program was used for elution: 60% eluent B (initial), 60–20% eluent B (from 0–7 min) and 20% eluent B (7–8 min). MS analysis was operated in positive mode with capillary voltage at 4.5 kV. The source temperature was kept at 350°C. Positively charged molecular ions of paraquat were monitored for precursor and products at *m*/*z* 186 to 171 and 155 [39]. For both methods, the mass spectrometer was operated under multiple reaction monitoring (MRM) mode. The unit mass resolution was used for ion mass analyzers. The enhanced product ion (EPI) scan rate was 1000 amu/s, and the scan range was 106 to 396 amu. Nitrogen gas was used as the collision and curtain gas, and argon gas was used as the nebulizer and heater gas. Empower® analyst software was used to control the LC/MS/MS system and acquire the data.

The limits of detection (LOD) for each toxin were 500 ppb for PCP and 2,4,6-TCP, 5 ppb for lindane and linuron, 12.5 ppb for diazinon, 0.1 ppb for trifluralin and 10 ppb for paraquat, with excellent reproducibility and sensitivity of the detection methods. Standard toxin solutions were spiked before and after 2 h of agitating, and the relative standard deviations (RSD) were <5%, showing a high recovery percentage and limited nonspecific binding. The detection methods were validated using standard calibration curves. Standard solutions of each toxin were individually prepared in mobile phase at concentration gradients between 25 ppm and 0.1 ppm to plot the standard curves. The standard curves of all toxins were linear (r^2^ > 0.99) between signal intensity and toxin concentration.

### 2.6. Data Calculations and Curve Fitting

The signal intensities for toxin by HPLC and LC/MS/MS, and UV/visible scanning spectrophotometry were used to calculate the free toxin in solution. The amount of toxin bound by clay at each data point was derived from the concentration difference between test and control groups and expressed as mol/kg on the isotherm. These data were then plotted using Table-Curve 2D and a computer program that was developed with Microsoft Excel to derive values for the variable parameters. The best fit for the data was a Langmuir model, which was used to plot equilibrium isotherms from triplicate analysis. The isotherm equation was entered as user-defined functions:

(2) $$\mathrm{Langmuir}\;\mathrm{model}\;q=Q_{\max}\left(\frac{K_{d}C_{w}}{1+K_{d}C_{w}}\right)$$

q = toxin adsorbed (mol/kg), Q_max_ = maximum capacity (mol/kg), K_d_ = distribution constant, C_w_ = equilibrium concentration of toxin.

Estimates for the Q_max_ and K_d_ were taken from the double-logarithmic plot of the isotherm. The plot displayed a break in the curve. The value on the x axis where the curve breaks is an estimate of K_d_^−1^. The value on the y axis where the curve breaks is an estimate of Q_max_ [28,40]. The Q_max_ was taken from the fit of the Langmuir model to the adsorption data. The definition of K_d_ was given by:

(3) $$K_{d}=\frac{q}{\left(Q_{\max}-q\right)C_{w}}$$

### 2.7. Hydra *Assay*

Hydra vulgaris were obtained from Environment Canada (Montreal, QC, Canada) and maintained at 18 °C in culture. The hydra classification method was used with modification [41] to rate morphology of the adult hydra as a quantitative indicator of solution toxicity. In this assay, the scoring of hydra morphology was objective and repeatable based on previous reports from our laboratory [42]. The assay included closely monitoring hydra morphology at short time intervals during the first two days (0, 4, 20, and 28 h) and at 24 h time intervals for the last three days (44, 68, and 92 h). Solutions were not changed during testing. The hydra response was scored and recorded after exposure to toxic chemicals, with and without sorbent inclusion. Mature and non-budding hydra, of similar size, were chosen for testing in order to minimize any potential differences related to dose between samples. Controls for this experiment included hydra media, with or without sorbent. Toxin treatment groups consisted of a pesticide mixture (2 ppm of each pesticide in hydra media) that was equivalent to a minimum effective dose that resulted in 100% mortality of hydra in 92 h. All test solutions were capped and prepared by shaking at 1000 rpm for 2 h and centrifugation at 2000 *g* for 20 min prior to exposure of hydra in Pyrex dishes. For each sample, three hydra were included into 4 mL of test media and maintained at 18 °C. The score, or average toxicity rating, was determined by calculating the average score for morphological changes in a group at a specific time point.

### 2.8. Statistical Analysis

A two way *t*-test was used to calculate statistical significance. Each experiment was independently triplicated to derive an average and standard deviation. In the *t*-test, the average COLE (coefficient of linear expansibility) ratio from COLE experiments, Q_max_ from equilibrium isothermal analyses and toxicity scores from the hydra assay were included to calculate D = control-test groups and D^2^. Then the t-value was calculated using the following equation (N = 3):

(4) $$t=\frac{\frac{\sum D}{N}}{\sqrt{\frac{\sum D2-\frac{\left(\sum D\right)2}{N}}{\left(N-1\right)N}}}$$

The t-value and degrees of freedom were compared in a *p*-value table to determine the statistical significance. Results were considered significant at *p* ≤ 0.05.

## 3. Results

### 3.1. Coefficient of Linear Expansibility in Water

**Figure 2.**
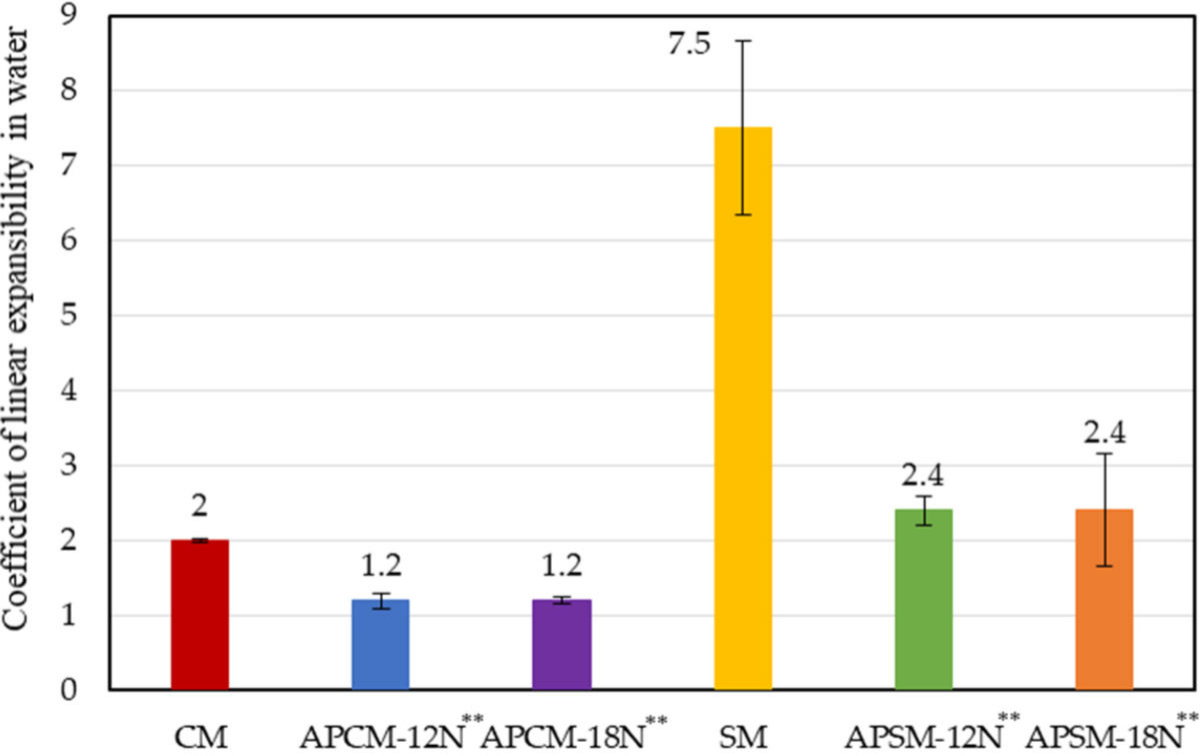
Coefficient of linear expansibility (COLE) for sorbents in water. The COLE value for parent sodium montmorillonite (SM) indicated significant hydration and expansibility, whereas COLE values for acid-processed calcium and sodium montmorillonites (APCMs and APSMs) displayed limited and decreased hydration energy and expansibility compared to parent calcium montmorillonite (CM) and SM clays (** *p* ≤ 0.01).

The COLE ratio indicates the expansibility of sorbents in water. COLE = expansion volume of clay/original volume of clay. The higher the ratio, the more expansion and hydration of the sample. The accuracy of this experiment was confirmed for parent CM and SM clays (COLE = 2 and 7.5, respectively). These values predicted limited swelling for CM and major swelling for SM (Figure 2). Compared to CM and SM, APCMs and APSMs displayed a significantly decreased COLE value (*p* ≤ 0.01), following acid treatment. This resulted from the significant replacement of cations in the interlayer with hydrogen ions and the leaching of ferric, ferrous, aluminum and magnesium ions which significantly alters the structure of clay layers.

### 3.2. Surface Area

**Figure 3.**
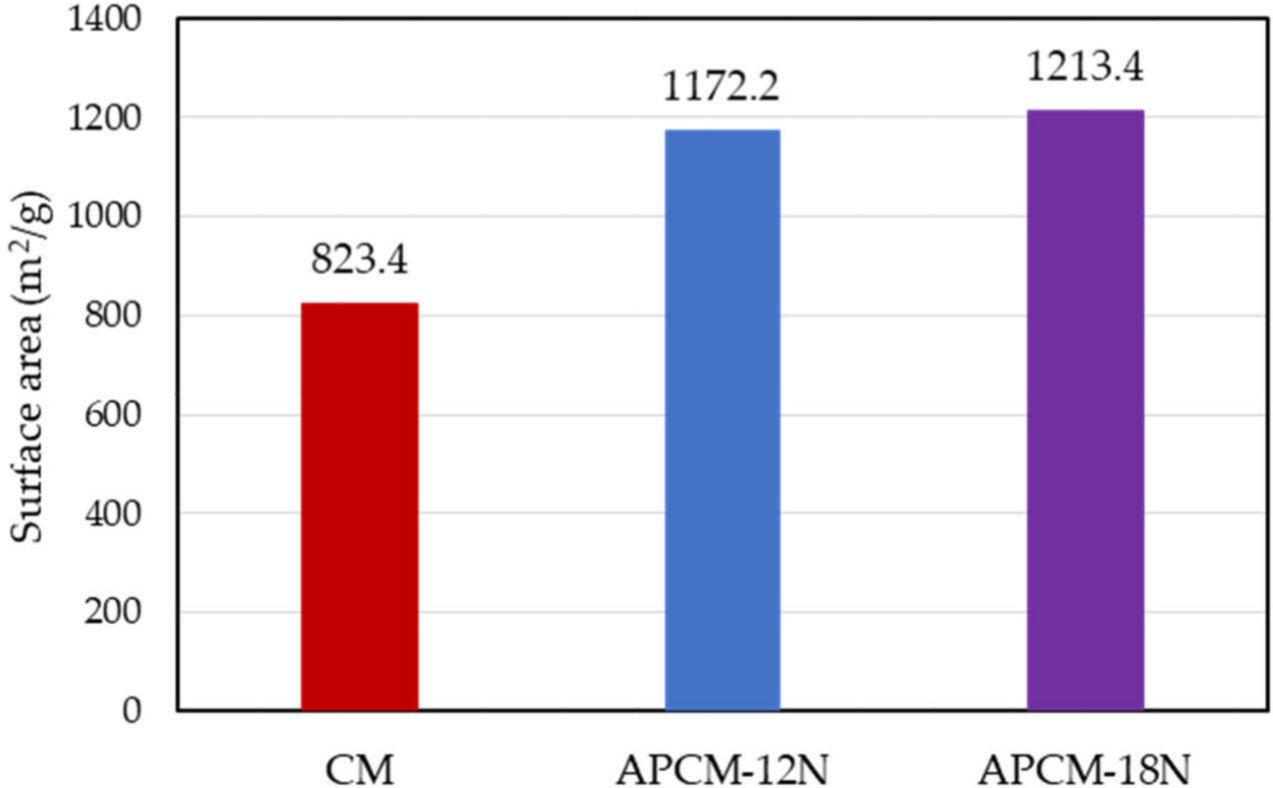
Surface area of parent CM and APCM determined by ethylene glycol absorbance onto clay surfaces.

APCMs exhibited higher total surface areas of 1172.2 m^2^/g for APCM-12N and 1213.4 m^2^/g for APCM-18N, indicating 42.4% and 47.4% increase compared to the parent CM, as shown in Figure 3. This result is also supported by earlier work with a similar CM clay [28].

### 3.3. Trace Metals

**Figure 4.**
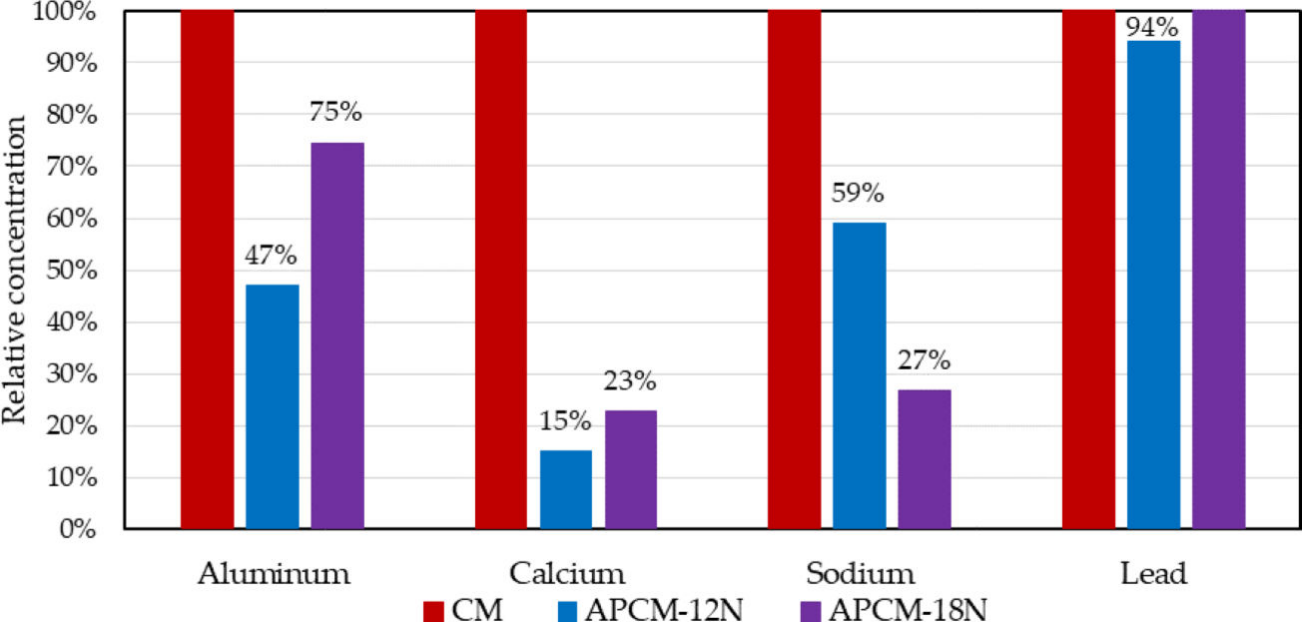
Relative values of trace metals in APCM compared to the parent CM clay.

In CM and APCM clays, calcium was the primary interlayer cation, while aluminum and sodium were present in the interlayer and in di-octahedral and tetrahedral sheets. The results in Figure 4 showed that both acid treatments decreased relative concentrations of aluminum, calcium and sodium compared to the parent clay. Treatment with 12N decreased aluminum and calcium by 53% and 85%, respectively, and 18N decreased sodium by 73%. The lead level in parent clay was detected as 11.7 ppm (the relative value was adjusted to 100%). Our results confirm that lead was bound tightly to the framework and lead concentration in the parent clay was not significantly changed by high level sulfuric acid treatment for 24 h at high temperature.

### 3.4. Adsorption Analyses

**Figure 5.**
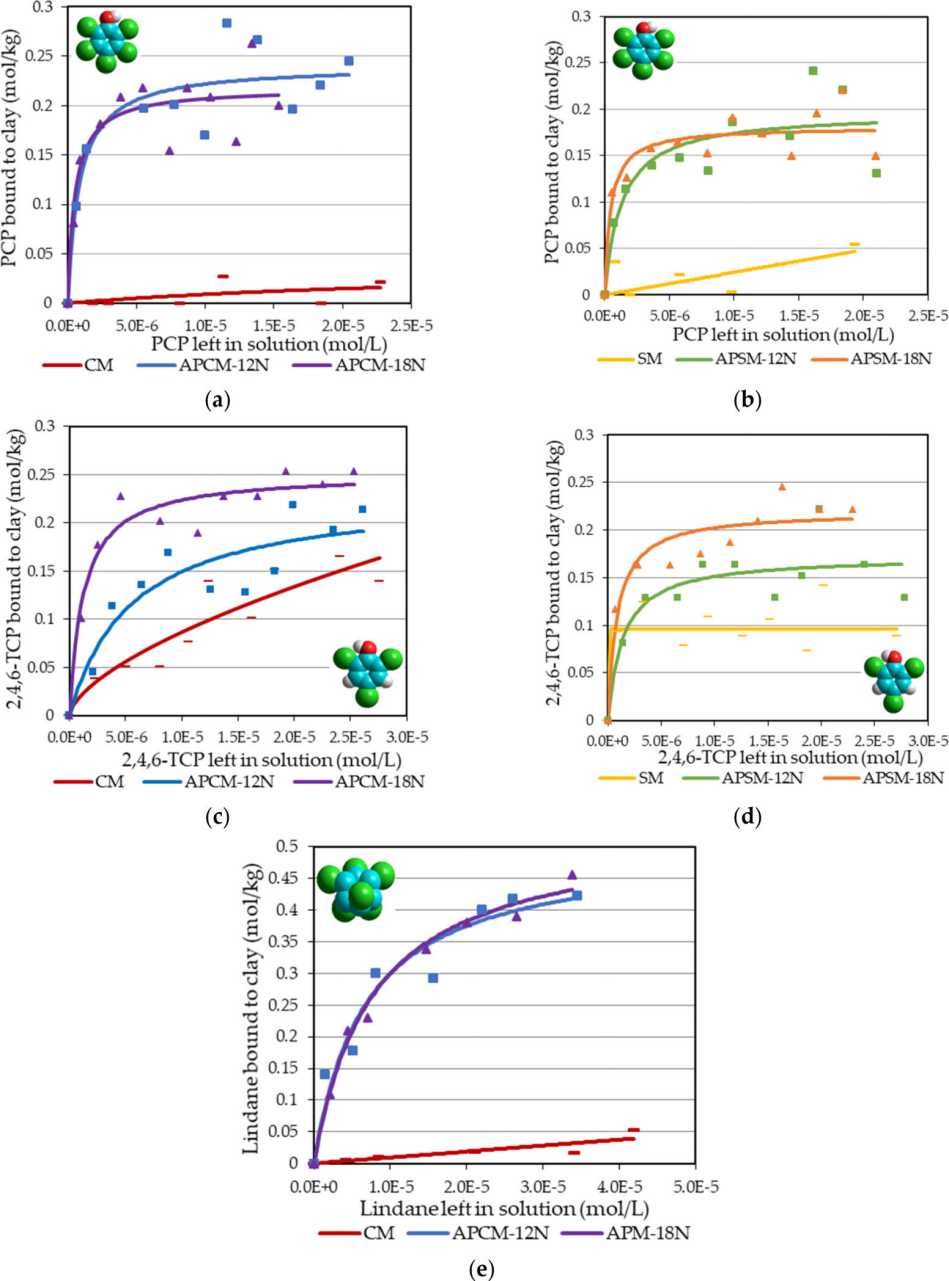
Langmuir adsorption isotherms for organochlorine pesticides: PCP, 2,4,6-TCP and lindane onto (**a**,**c**,**e**) APCM and (**b**,**d**) APSM surfaces, versus parent montmorillonites. Top: (**a**,**b**) showing isotherms for PCP. Middle: (**c**,**d**) showing isotherms for 2,4,6-TCP. Bottom: (**e**) showing isotherm for lindane.

Isothermal analyses of the sorption of organochlorine pesticides, including PCP, 2,4,6-TCP and lindane, onto APM surfaces are shown in Figure 5. The plots of APMs fit the Langmuir model (r^2^ > 0.80) as indicated by good correlation coefficients and curved shapes indicating the presence of saturable sites. The plots of parent CM and SM showed a partitioning trend that fit the Freundlich model indicating the lack of a saturable Q_max_. Compared to parent clays, acid treatment resulted in a Q_max_ in the range of 0.2 mol/kg for the chlorophenol toxins and approximately 0.5 mol/kg for lindane. Adsorption isotherms of APCMs and APSMs showed similarities in Q_max_ and K_d_ values for PCP and 2,4,6-TCP.

**Figure 6.**
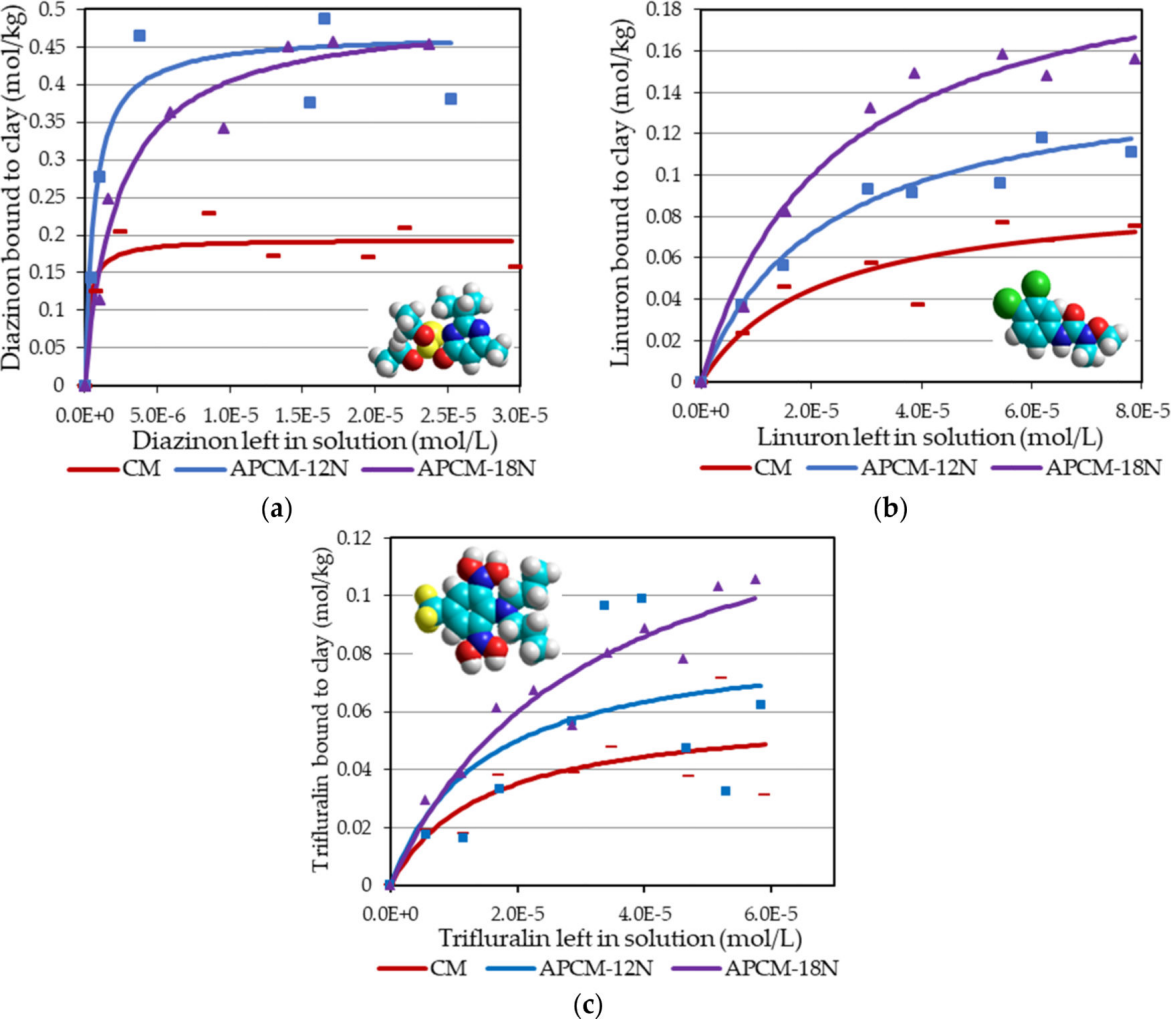
Langmuir adsorption isotherms for (**a**) diazinon (organophosphate), (**b**) linuron (urea-type) and (**c**) trifluralin (dinitroaniline) onto APCM surfaces.

**Figure 7.**
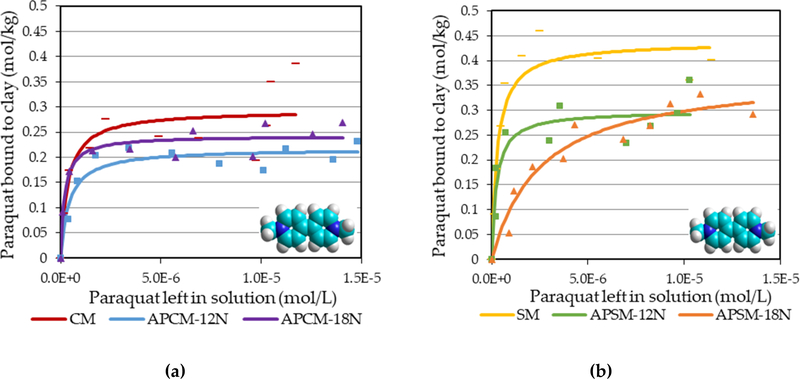
Langmuir adsorption isotherms for paraquat (bipyridyl) onto (**a**) APCM and (**b**) APSM surfaces.

Similarly, isothermal adsorption plots in Figure 6 showed sorption patterns for other pesticides including diazinon, linuron and trifluralin that fit the Langmuir model (r^2^ > 0.95). APCMs significantly increased the binding capacity of diazinon (~0.5 mol/kg), linuron (~0.2 mol/kg) and trifluralin (~0.1 mol/kg) compared to that of parent CM clay. Paraquat has two permanent positive charges on the quaternary nitrogens and these charges facilitate its attraction to the negatively charged clay interlayer surfaces of CM and SM (Q_max_ = 0.29 and 0.44 mol/kg, respectively) as indicated by the Langmuir model (r^2^ > 0.94); due to less negatively charged interlayers in acid processed clays, the adsorption of paraquat by APCMs and APSMs was slightly reduced, as expected (Figure 7). The sorption parameters for each toxin on APMs and parent clays are summarized in Table 1.

**Figure 8.**
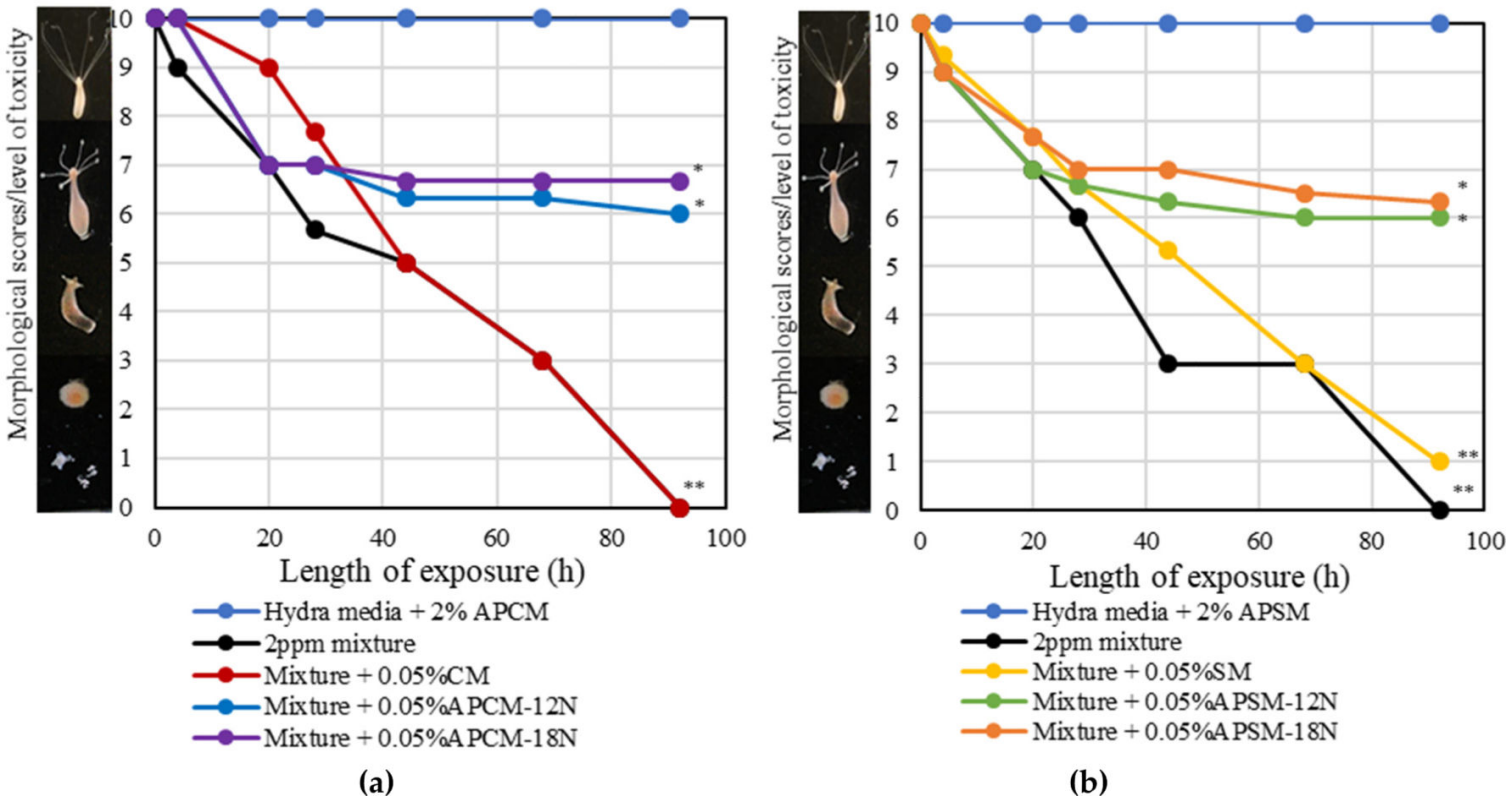
Hydra toxicity from a pesticide mixture (2 ppm/pesticide) and protection by (**a**) APCM and (**b**) APSM versus parent montmorillonite clays with an inclusion of 0.05% sorbent. Hydra media with 2% APMs and toxin controls are included for comparison. APM sorbents significantly protected against the pesticide mixture with 60–67% protection at the end point (92 h). CM and SM showed limited protection (10%) at the same inclusion rate (* *p* ≤ 0.05, ** *p* ≤ 0.01).

To investigate the safety and binding efficacy of APMs, a design mixture of pesticides (at an equal concentration of 2 ppm for each toxin) was prepared and its toxicity was confirmed in a living organism (Hydra vulgaris). This pesticide design mixture resulted in 100% mortality of hydra, following 92 h exposure. The inclusion of APCMs and APSMs at a rate of only 0.05% resulted in significant protection of hydra ranging from 60% to 67%. The inclusion of parent CM and SM clays, at the same inclusion rate, resulted in 0 and 10% protection, respectively. Importantly, APM inclusion at levels as high as 2% w/v in the hydra media showed no effect on hydra (Figure 8).

## 4. Discussion

Previously, montmorillonite clays have been shown to be safe for human and animal consumption based on numerous animal models and human intervention studies in Africa and the United States. However, these parent clays showed a preference for the adsorption of aflatoxins and positively charged chemicals. To develop broad-acting sorbents that can reduce exposures to diverse mixtures of environmental chemicals, we have treated montmorillonites with acid to increase their surface area and porosity, resulting in more active sites for toxin sequestration and binding. The safety of broad-acting APMs and their increased efficacy for aflatoxins, zearalenone, glyphosate, and PCBs have been previously described [24,25]. This study was designed to investigate the binding efficacy of APMs for widely used and highly toxic pesticides (individuals and mixtures) with different structural morphologies and modes of toxic action.

Based on our results, acid treatment was shown to significantly decrease expansibility in water (*p* ≤ 0.01), increase surface area by almost 50% compared to the parent clay, and decrease the concentration of trace metals, including framework aluminum, and interlayer calcium and sodium. This has been shown to enhance mesoporosity and toxin-binding activity in APMs [43–45]. Our results are in alignment with Fourier transform infrared (FTIR) spectroscopy and scanning electron microscopy (SEM) reports on the acid treatment of clays [27,46]. The lead level in APMs showed that lead concentration was not changed by sulfuric acid treatment for 24 h at high temperature. This result suggests that lead is very tightly bound within the clay structure, and cannot be significantly dissociated, even in extreme conditions such as heat and strong acid treatment for long durations. Importantly, these results suggest that lead should not be significantly bioavailable from this clay following ingestion by humans and animals. This conclusion is further supported by our earlier animal and human intervention studies, that showed no significant increase in serum lead levels following ingestion of CM clay as high as 2.0% in the diet [47].

Adsorption isothermal analyses of organochlorine pesticides (PCP, 2,4,6-TCP and lindane) in Figure 5 showed that APCMs and APSMs were able to stably bind these toxins based on Langmuir plots that indicated saturable binding sites, whereas parent clays fit a Freundlich model indicating partitioning and weak binding. The binding capacities for APMs were approximately 0.2 mol/kg for PCP and 2,4,6-TCP and 0.5 mol/kg for lindane. APCMs and APSMs showed similarities in pesticide binding efficacy, suggesting that the predominant interlayer cations (calcium or sodium) have been replaced by protons from the acid. Adsorption isotherms in Figure 6 showed that APCMs can also serve as effective sorbents for other types of pesticides, such as diazinon, linuron and trifluralin. Binding of these toxins to APCMs significantly increased Q_max_ compared to parent CM (*p* ≤ 0.05).

APCMs and APSMs showed a slightly decreased Q_max_ for paraquat (Q_max_ = 0.21 and 0.24 mol/kg for APCMs; 0.3 and 0.37 mol/kg for APSMs, respectively) compared to parent CM and SM (Q_max_ = 0.29 mol/kg for CM; 0.44 mol/kg for SM, respectively). The two positively charged nitrogens on the bipyridine rings of paraquat can facilitate the toxin’s attraction to the negative interlayer surfaces on parent CM and SM. The small decrease in paraquat adsorption could also be attributed to the electrostatic repulsion between the positively charged surface of acid-activated clays and the positively charged paraquat molecule, in addition to the competitive adsorption between the H^+^ ions and paraquat cations to reach the surface [46]. Additionally, decrease in intact (negatively charged) interlayer surfaces in APMs could also contribute to less adsorption of paraquat.

The final product of APMs consists of a mixture of delaminated parent clay layers containing chains of amorphous silica on the edges, amorphous silica, and cross-linked silica [25,26,46]. This polymorphic structure with diverse binding sites, higher surface area and enhanced porosity compared to the parent clays, can contribute to the increased binding efficacy for diverse chemicals observed in this study. The reduction in interlayer surfaces in the APM clays can result in a lower binding efficacy for positively charged substances versus clays with intact interlayers. This finding is in alignment with our earlier study with glyphosate. The adsorption of zwitterionic glyphosate at pH 7, where it had an overall negative charge, showed a higher binding efficacy for APMs versus parent montmorillonites. Glyphosate at pH 2, however, had a net positive charge and thus was shown to be bound more effectively onto parent montmorillonites than APMs [48]. Therefore, APMs are more effective in adsorbing organophilic, neutral and negatively charged toxins and can be mixed with parent montmorillonites for binding of toxins that are positively charged.

To investigate the safety and binding efficacy of APMs, a mixture of these pesticides (2 ppm/pesticide) was exposed to hydra. Hydra vulgaris is very sensitive to environmental toxins and has been widely used as a sensitive indicator of toxicity. In this study, the pesticide mixture resulted in 100% mortality of hydra following exposure for 92 h. APCMs and APSMs, at a very low inclusion of 0.05% *w*/*v*, showed significant protection (60% to 67%) against the pesticide mixture. Parent CM and SM clays, at the same inclusion rate, only showed 0% and 10% protection, respectively. The hydra assay showed the increased binding ability of APMs compared to parent montmorillonites and established the safety of APMs at inclusion levels as high as 2% *w*/*v*. It is possible that APMs at higher inclusion will result in more significant protection against toxicity based on previous dosimetry studies [47,49,50]. Importantly, we have used this organism to select optimal sorbents for food-borne and environmental toxicants, prior to safety and efficacy studies in animals and humans. In these studies, the hydra assay has significantly confirmed our in vitro, in vivo, and in silico results. Rodent assays are warranted to further validate the sorption efficacy of APMs and their ability to reduce toxicant exposures in humans and animals.

The main novelty of this study is the fact that we can utilize APMs that contain surface areas and porosities higher than parent clays as broad-acting toxin enterosorbents for multiple pesticides from major classes, thus reducing human and animal exposure during disasters and emergencies. In this study, novel APMs were shown to significantly limit the expansibility and swelling in water, increase surface area and reduce trace metals. Adsorption isotherms suggest that APMs were able to bind highly toxic pesticides with high binding capacity, affinity and tightness of sorption compared to parent clays. APMs especially enhanced the binding for neutral and negative pesticides and may be mixed with parent montmorillonites to facilitate the binding of positive substances (like paraquat). This is the first report of a sorbent material (other than activated carbon) with high binding efficacy for diverse pesticides. The in vivo hydra assay showed that APMs were safe for hydra at 2% inclusion rate, and its inclusion at 0.05% resulted in a significant protection of hydra against a toxic mixture of pesticides. It is possible that these, and other acid processed clays, may be broad-acting in their ability to decrease exposures to other pesticides and hazardous environmental contaminants. We anticipate the short-term inclusion of broad-acting APMs in the diet of humans and animals as a protective measure to decrease unintended exposures from contaminated food and water supplies at the site of disasters such as hurricanes, floods, chemical spills, fires, and acts of terror.

## Figures and Tables

**Table 1.** Summary of sorption parameters for APMs and parent montmorillonites.

Sorbents	PCP	2,4,6-TCP	Lindane	Diazinon	Linuron	Trifluralin	Paraquat
CM	K_d_ = 2.7 × 10^4^	Kd = 4 × 10^2^	K_d_ = 2.8 × 10^2^	Q_max_ = 0.19 K_d_ = 3.6 × 10^6^	Q_max_ = 0.09 K_d_ = 4.7 × 10^4^	Q_max_ = 0.06 K_d_ = 6.9 × 10^4^	Q_max_ = 0.29 K_d_ = 2.8 × 10^6^
SM	K_d_ = 5.1 × 10^2^	Kd = 5 × 10^20^	-	-	-		Q_max_ = 0.44 K_d_ = 3.4 × 10^6^
APCM-12N	Q_max_ = 0.24 K_d_ = 1.2 × 10^6^	Q_max_ = 0.23 K_d_ = 1.8 × 10^5^	Q_max_ = 0.5 K_d_ = 1.5 × 10^5^	Qmax = 0.47 ** K_d_ = 1.6 × 10^6^	Qmax = 0.15 * K_d_ = 4.5 × 10^4^	Q_max_ = 0.09 K_d_ = 6.9 × 10^4^	Q_max_ = 0.21 K_d_ = 2.4 × 10^6^
APSM-12N	Q_max_ = 0.2 K_d_ = 7.8 × 10^5^	Q_max_ = 0.17 K_d_ = 7.1 × 10^5^	-	-	-		Q_max_ = 0.3 * K_d_ = 4.1 × 10^6^
APCM-18N	Q_max_ = 0.22 K_d_ = 1.9 × 10^6^	Q_max_ = 0.25 K_d_ = 8.1 × 10^5^	Q_max_ = 0.53 K_d_ = 1.3 × 10^5^	Qmax = 0.5 ** K_d_ = 4.3 × 10^5^	Qmax = 0.22 ** K_d_ = 4.2 × 10^4^	Qmax = 0.15 * K_d_ = 3.2 × 10^4^	Q_max_ = 0.24 K_d_ = 5.1 × 10^6^
APSM-18N	Q_max_ = 0.18 K_d_ = 2.2 × 10^6^	Q_max_ = 0.22 K_d_ = 1.2 × 10^6^	-	-		-	Q_max_ = 0.37 K_d_ = 3.9 × 10^5^

(CM, calcium montmorillonite; SM, sodium montmorillonite; APCM, acid processed calcium montmorillonite; APSM, acid processed sodium montmorillonite; Q_max_, binding capacity; K_d_, binding affinity; * *p* ≤ 0.05; ** *p* ≤ 0.01).
